# 

**Published:** 1871-01

**Authors:** 


Vol. XII. JANUARY, 1871. No. 1.

### CHICAGO Medical Examiner,

N. S. DAVIS, M.D., Editor,

AND

F. H. Davis, Assist.-Editor.

TERMS, $3.00 PER ANNUM, IN ADVANCE.

CHICAGO:
ROBERT FERGUS' SONS, PRINTERS,
12 CLARK AND 242—248 ILLINOIS STREET
1871.

